# Combatting *Pseudomonas aeruginosa* with β-Lactam Antibiotics: A Revived Weapon?

**DOI:** 10.3390/antibiotics14050526

**Published:** 2025-05-20

**Authors:** Dylan W. Zhao, Christopher T. Lohans

**Affiliations:** Department of Biomedical and Molecular Sciences, Queen’s University, Kingston, ON K7L 3N6, Canada; dylan.zhao@queensu.ca

**Keywords:** antibiotic resistance, *Pseudomonas aeruginosa*, β-lactam antibiotics, adjuvant agents

## Abstract

*Pseudomonas aeruginosa* is a significant threat to public health as an aggressive, opportunistic pathogen. The use of β-lactam antibiotics such as penicillins, cephalosporins, monobactams, and carbapenems remains a front-line treatment against *P. aeruginosa*. However, the widespread use of β-lactams has led to the emergence of β-lactam-resistant isolates that significantly increase the economic burden and risk of mortality in patients. With the declining productivity of the antibiotic discovery pipeline, research has investigated synergistic agents to revive the use of β-lactam antibiotics against β-lactam-resistant *P. aeruginosa*. In this review, we summarize the mechanism of β-lactam antibiotics and provide an overview of major mechanisms associated with β-lactam resistance in *P. aeruginosa*. We then describe the background and use of three promising classes of agents that have shown extensive beneficial effects with β-lactam antibiotics against *P. aeruginosa*, namely β-lactamase inhibitors, bacteriophages, and antimicrobial peptides. The current understanding of the mechanisms of these synergistic agents is discussed. Lastly, we provide an overview of the current barriers impeding antibiotic development, and offer a glimpse into recent advances of artificial intelligence-based discovery that may serve as a new foundation for antimicrobial discovery and treatment.

## 1. Introduction

*Pseudomonas aeruginosa* is a Gram-negative bacillus that is a prevalent cause of nosocomial infections, and is responsible for mortality rates as high as 61% in ventilator-associated pneumonia, post-surgical infections, burn wounds, and cystic fibrosis-associated infections [1,2,3]. Epidemiological studies across international settings emphasize how *P. aeruginosa* remains a critical, global threat to public health. In a systematic analysis of global mortality from bacterial infections in 2019, *P. aeruginosa* was one of five pathogens responsible for 54.9% of the 7.7 million deaths analyzed, contributing to 590,000 deaths alone [4]. The 2016 EPINE survey found that *Escherichia coli* and *P. aeruginosa* were the most prevalent nosocomial pathogens in Spain, with *P. aeruginosa* accounting for 10.5% of all infections [5]. From European hospital-acquired infection reports between 2011 and 2021, *P. aeruginosa* caused approximately 9% of all infections as the fourth most common nosocomial pathogen [6]. In North America, the Centers for Disease Control and Prevention found that *P. aeruginosa* accounted for 7.1% of all nosocomial infections [3].

Once a person is infected by *P. aeruginosa*, this bacterium aggressively employs various virulence factors (e.g., toxin secretion systems, outer membrane vesicles, rhamnolipids) to enhance colonization, directly cause tissue damage (especially in the pulmonary tract), and evade the immune response [7,8,9,10,11,12,13,14,15,16,17,18]. As *P. aeruginosa* infections can rapidly progress towards systemic bacteremia, sepsis, and death [19], having adequate antibiotic regimens available remains imperative. Following treatment guidelines published by the Infectious Diseases Society of America and Health Canada, the use of β-lactam antibiotics (e.g., penicillins, cephalosporins, monobactams, and carbapenems) remains the first line of treatment for *P. aeruginosa* with recommended agents including ceftazidime, cefepime, piperacillin, meropenem, and imipenem [20]. However, with β-lactam prescriptions representing over 65% of the antibiotic global market [21,22,23], combined with the improper use, disposal, and overall stewardship of antibiotics [24], resistant *P. aeruginosa* strains have been identified worldwide [25,26,27,28]. Although the extent of β-lactam resistance on a global scale is unknown, a 2-year analysis of multi-drug resistant (MDR) Gram-negative infections in a Greek tertiary hospital revealed 55% of all *P. aeruginosa* isolates from the intensive care unit were resistant to carbapenems, a β-lactam often reserved as a last resort [29]. As resistant *P. aeruginosa* infections can cause an approximate two-fold increase in the risk of mortality and a 30% increase in cost relative to susceptible infections, this microorganism remains a significant financial burden and threat to public health [30,31].

Although resistance against β-lactams remains a constant threat, sustained efforts have not only yielded novel antibiotics with a β-lactam scaffold, but have also contributed to the development of novel agents that have been shown to enhance or rescue the efficacy of β-lactam antibiotics. These recent successes not only relieve some pressure on the deteriorating antibiotic discovery pipeline, but also offer a new avenue of therapies to treat MDR *P. aeruginosa*. As several excellent reviews already exist on combination therapies of β-lactams and other antibiotics [32,33,34,35,36], this review examines the current literature describing three prominent classes of potential adjuvant agents: β-lactamase inhibitors, bacteriophages, and antimicrobial peptides. We begin by describing the mechanism of β-lactam antibiotics and the β-lactam resistance mechanisms that occur in *P. aeruginosa*. We next describe the mechanism and use of synergistic adjuvants for the rescue of β-lactam activity. Finally, we provide a brief perspective on the future outlook relating to the use of machine learning to develop novel agents to combat β-lactam-resistant *P. aeruginosa* infections.

## 2. β-Lactam Antibiotics and Mechanisms of Resistance

### 2.1. Mechanism and Classification of β-Lactams

**Figure 1:**
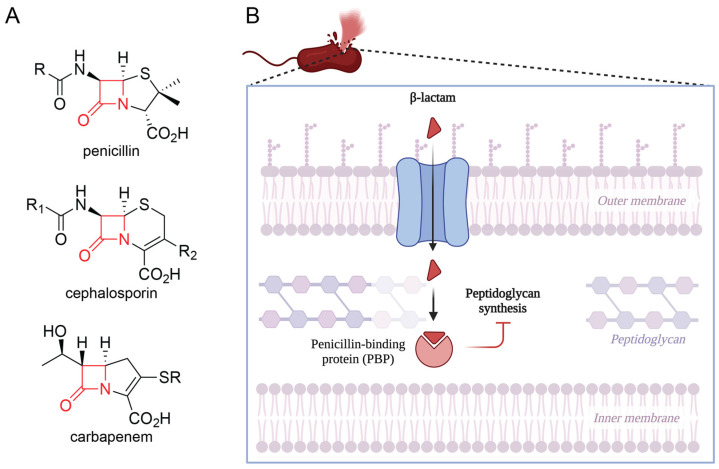
Overview of β-lactam structure and activity. (**A**) Representative chemical structures of β-lactams belonging to the penicillin, cephalosporin, and carbapenem subclasses. The core β-lactam ring is indicated in red. (**B**) β-lactam antibiotics can cross the Gram-negative outer membrane through porin proteins embedded in the membrane, thereby accessing the periplasmic space that contains PBPs. The bactericidal effect of β-lactams against *P. aeruginosa* involves the inhibition of PBP3, which normally catalyzes the formation of peptide cross-links in peptidoglycan. Inhibition of PBP3 and the subsequent decreased formation of peptidoglycan cross-links significantly weakens the cell wall and induces autolysis. Created in BioRender (https://www.biorender.com/).

β-lactam antibiotics are classified based on their core, four-membered β-lactam ring (Figure 1A), the structural motif that is essential for their bactericidal activity [37]. This scaffold is conserved across all four major classes of β-lactam antibiotics (penicillins, cephalosporins, carbapenems, monobactams) and is fused to an additional heterocycle in all classes with the exception of the monobactams [38]. Subtle changes in the functional groups decorating this core give rise to distinct differences in overall biological activity and interactions with bacterial targets and resistance mechanisms. As the carbapenem subclass is frequently reserved as an antibiotic of last resort [39], resistance against carbapenems remains a significant threat to public health and will be a focus of this review.

To withstand fluctuations in osmotic stress, bacteria rely on the structural integrity of peptidoglycan in the cell wall. This structural integrity is greatly dependent on cross-links between peptide chains in peptidoglycan that are formed by penicillin-binding proteins (PBPs) [21,40]. In Gram-negative bacteria such as *P. aeruginosa*, peptidoglycan and some of the enzymes that synthesize it are found in the periplasmic space between the outer and cytoplasmic membranes. β-lactam antibiotics in the periplasm interfere with cell wall synthesis by targeting PBPs through a suicide inhibition mechanism (Figure 1B). This is achieved through an acylation reaction between the β-lactam ring and an essential serine residue in the PBP active site, forming a stable acyl–enzyme complex that blocks PBP-catalyzed cross-link formation [41]. Consequently, the bacterial cell wall weakens and becomes increasingly susceptible to osmotic stress, leading to cellular autolysis [21,40].

### 2.2. β-Lactamase-Mediated Resistance

**Figure 2:**
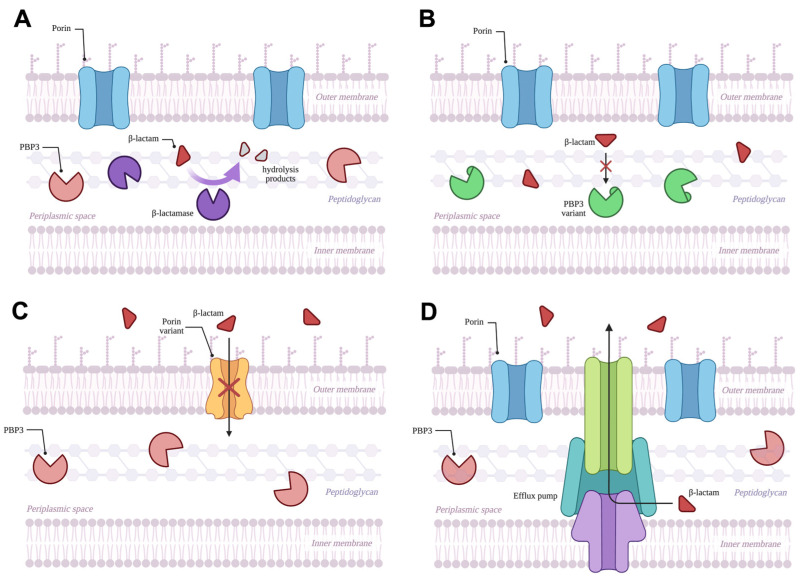
Overview of β-lactam resistance mechanisms in *P. aeruginosa*. (**A**) β-lactamase enzymes in the periplasmic space degrade β-lactams, preventing them from inhibiting PBPs such as PBP3. (**B**) Certain mutations to the *ftsl* gene modify the structure of PBP3 such that β-lactam antibiotics are no longer able to bind and interfere with peptidoglycan synthesis. (**C**) Mutations to the genes encoding for porins such as OprD and OpdP can decrease the entry of β-lactams into the periplasmic space by reducing the porin content in the outer membrane or by modifying the structures of porins. (**D**) Increased expression of efflux pumps such as MexAB-OprM allows *P. aeruginosa* to expel β-lactam antibiotics from the periplasm, preventing them from interacting with PBPs. Created in BioRender.

A prominent resistance mechanism employed by *P. aeruginosa* and other β-lactam-resistant Gram-negative bacteria is the production of β-lactamases. These enzymes catalyze a hydrolytic reaction that opens the β-lactam ring, rendering the antibiotic unable to inhibit PBPs (Figure 2A). In Gram-negative bacteria such as *P. aeruginosa*, β-lactamases primarily reside in the periplasmic space. β-lactamases are commonly categorized according to their sequence motifs and mechanisms into classes A, B, C, and D in the Ambler classification system (Table 1) [42]. β-lactamases can also be more broadly differentiated based on their mechanisms of catalysis; the serine β-lactamases (SBLs; Ambler classes A, C, and D) rely on a covalent, two-step hydrolytic mechanism mediated by a nucleophilic serine residue, while metallo-β-lactamases (MBLs; Ambler class B) hydrolyze β-lactams using a water molecule activated by one or more zinc ions. To this point, at least 120 different β-lactamases have been identified in clinical isolates of *P. aeruginosa* [43], and these enzymes remain clinically significant given their ability to confer high levels of resistance against β-lactams and their horizontal mobility, as their genes are commonly carried on plasmids. β-lactamases are also often categorized based on their substrate specificities, yielding groups such as the penicillinases, cephalosporinases, extended-spectrum β-lactamases (ESBLs), and carbapenemases. As carbapenems are reserved as antibiotics of last resort due to their ability to withstand hydrolysis from most β-lactamases, carbapenemase-producing organisms are widely regarded as one of the greatest threats resulting from antibiotic resistance.

Within the Ambler classification system, hundreds of carbapenemase variants fall under classes A, B, and D [48]. Prevalent carbapenemases within these respective classes include the KPC family (class A; SBLs), the NDM, IMP, and VIM families (class B; MBLs), and the OXA family (class D; SBLs). As many reviews have been published on the Ambler classification, epidemiology, and catalytic spectra of β-lactamases [41,44,45,49,50,51,52], this section will highlight the clinical consequences of SBL- and MBL-producing *P. aeruginosa*.

The KPC family of SBLs can hydrolyze all classes of β-lactams, while the OXA family, although sharing a similar spectrum of hydrolysis, exhibits significantly reduced activity against carbapenems while still contributing to carbapenem resistance in vivo [53]. These properties complicate the clinical detection of OXA-type carbapenemases and have earned them a reputation of being a “phantom menace” [54,55,56,57]. Many MBLs are carbapenemases that can hydrolyze most β-lactams with the notable exception of monobactams like aztreonam [58]. To date, at least 32 variants of IMP and 23 variants of VIM have been identified in *P. aeruginosa* [59], and the reported prevalence of these enzymes is as high as 30% among carbapenem-resistant isolates [60]. Due to the broad catalytic activities of the MBLs, the presence of resistance genes to other antibiotics within the same plasmid can lead to pan-resistant phenotypes. Another significant challenge posed by MBLs is that, unlike SBLs, they are not targeted by any of the β-lactamase inhibitors that are currently available for clinical use due to the drastic differences in mechanisms of catalysis between MBLs and SBLs [61].

Clinically, the presence of class A, B, and D carbapenemases has been consistently shown to complicate treatment efficacy and increase *P. aeruginosa* infection mortality [62,63,64,65,66,67,68,69,70,71,72,73]. A report by Zhang et al. followed the spread of high-risk clones of carbapenemase-producing *P. aeruginosa* between 2020 and 2022 in Zhejiang, China [62]. Eight out of the 192 isolates were identified as belonging to the high-risk ST463 clone that co-harbored KPC-2 and AFM-1. Due to the presence of both class A and B carbapenemases, all eight isolates exhibited resistance against all β-lactams and β-lactam/β-lactamase inhibitor combinations tested, and were only susceptible to amikacin and intermediately susceptible to colistin [62]. In an analysis of carbapenem-resistant *P. aeruginosa* isolates from Qatar, almost all isolates (96%) possessed an OXA-family carbapenemase, with 26.7% of isolates carrying an MBL and 4% of isolates producing representatives of all four Ambler classes of β-lactamases [74]. Similar to previous reports, 40% of the MBL-producing isolates were susceptible to aztreonam due to the absence of ESBLs [74]. As a further illustration of the concern associated with the transmissibility of plasmid-encoded β-lactamases, all isolates were even resistant to β-lactam/β-lactamase inhibitor combinations that were not clinically available in Qatar at the time of study [74]. To highlight the morbidity associated with the production of β-lactamases in *P. aeruginosa*, the pan-resistance of a strain characterized by Costa-Júnior et al. was attributed in part to the production of KPC-2 and VIM-2 alongside the presence of the aminoglycoside resistance gene *rmtD1* [75]. Hence, preserving the efficacy of last-resort carbapenems through proper stewardship remains critical for public health in the reduction of highly morbid MDR *P. aeruginosa* infections [76].

### 2.3. Target Modification

Changes to the active sites of PBPs can interfere with the ability of β-lactams to bind and acylate the conserved nucleophilic serine residue (Figure 2B). In *P. aeruginosa*, the transpeptidase PBP3 (encoded by the *ftsl* gene) catalyzes the formation of peptidoglycan cross-links and is essential for bacterial growth [77]. The loss of β-lactam efficacy in the presence of *ftsl* mutations, particularly those impacting the active site, has been extensively studied in experimental models and clinical isolates of *P. aeruginosa* [78,79]. Given that the binding interactions between the different classes of β-lactam antibiotics and the PBP3 active site differ, changes to the active site can confer varying degrees of β-lactam resistance. Moreover, a change to PBP3 can lead to different interactions with β-lactams belonging to the same class. For example, PBP3 variants observed in *Pseudomonas* isolates from a cystic fibrosis patient treated with β-lactams possessed two notable amino acid substitutions, V465G and A244T, with the former conferring resistance to both aztreonam (monobactam) and cefsulodin (cephalosporin) and the latter was associated with ceftazidime (cephalosporin) and piperacillin (penicillin) resistance [80]. Another study in adult cystic fibrosis patients illustrated that production of the PBP3 R504C variant conferred resistance to ceftazidime and cefsulodin, whereas the P527S variant was associated with resistance to aztreonam, cefepime, ceftazidime, and cefsulodin [81].

### 2.4. Decreased Membrane Permeability: Role of Porins

Porins are β-barrel proteins that mediate the passive entry of hydrophilic compounds (including β-lactam antibiotics) across the outer membranes of Gram-negative bacteria [82,83]. As such, mutations that cause changes to porin structure or to the number of porins in the outer membrane can protect bacteria from β-lactam activity (Figure 2C). While not all 26 porins identified in *P. aeruginosa* are involved in β-lactam uptake, those belonging to the OprD and Opd (P/D/H) families have been investigated for their role in β-lactam resistance [84]. In addition to small basic amino acids and peptides, the OprD porin is a crucial mediator in the uptake of carbapenems in *P. aeruginosa* [85,86,87,88]. Point mutations, premature stop codons, and frameshifts in the *oprD* gene have been shown to reduce or abolish OprD expression and lead to carbapenem resistance [88]. Of note, mutations to *oprD* were not observed to impact susceptibility to other β-lactam antibiotics (e.g., penicillins, cephalosporins, and monobactams) [89,90,91]. Regarding the Opd family of porins, the loss of OpdP was shown to reduce susceptibility to meropenem and, in an *opdD* knockout strain, to imipenem and doripenem as well, supporting that OpdD facilitates the entry of carbapenems in *P. aeruginosa* [91]. The loss of OpdH was also shown to reduce susceptibility to ceftazidime but not other cephalosporins [89,90,91,92].

### 2.5. Efflux Pumps

Efflux pumps are transmembrane proteins that can facilitate the expulsion of various substances (including β-lactam antibiotics) from the periplasm across the outer membrane and into the extracellular environment (Figure 2D) [93], with many of the pumps that contribute to antibiotic efflux belonging to the resistance-nodulation-division (RND) family [94]. Structurally, these RND pumps are a tripartite system comprising a periplasmic adaptor protein, a transmembrane inner-membrane transporter, and a protein channel through the outer membrane [95,96]. In *P. aeruginosa*, efflux pumps MexAB-OprM, MexXY-OprM, MexCD-OprJ, and MexEF-OprN have been shown to contribute to β-lactam resistance, with each exporting a particular selection of β-lactams (Table 2). Unsurprisingly, overexpression of these pumps has been identified in many β-lactam-resistant *P. aeruginosa* clinical isolates [85,86,97,98,99,100,101,102,103], with one multicenter study in France revealing efflux pump overexpression in 88% of isolates collected from cystic fibrosis patients [85]. Specifically, 71 out of 80 isolates overexpressed at least one or more efflux system, with 65 overexpressing MexXY-OprM, 36 overexpressing MexAB-OprM, and two overexpressing MexCD-OprJ [85]. Within the overexpressing isolates, an additional 29 and five simultaneously overexpressed two or three efflux systems, respectively [85]. The molecular basis of efflux pump overexpression is often attributed to mutations in repressor genes that regulate each respective *mex* operon. As one example of many [104,105,106,107], overexpression of MexAB-OprM is primarily attributed to loss of function mutations in the DNA-binding region of the MexR repressor protein [108], preventing MexR from repressing the *mexAB oprM* operon [109,110]. Such mutations were identified in clinical isolates resistant to meropenem, ceftazidime, aztreonam, ticarcillin, and carbenicillin [111,112]. Deletions in repressor genes *nalD* and *nalC* also contribute to MexAB-OprM overexpression, although these changes are less prevalent [111,113].

## 3. Adjuvant Agents: Mechanisms and Use

### 3.1. β-Lactamase Inhibitors

**Figure 3:**
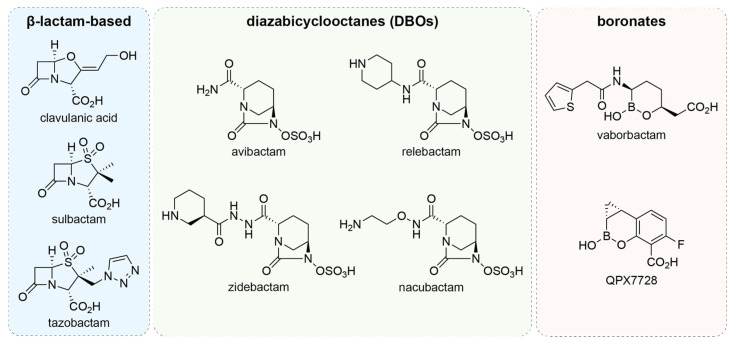
Representative β-lactamase inhibitors. Chemical structures of selected β-lactamase inhibitors that are in current use or under clinical development, organized according to their scaffolds as β-lactams, diazabicyclooctanes, and boronates.

Since the discovery of clavulanic acid in 1979 as an irreversible inhibitor of certain class A β-lactamases [117], the development of novel β-lactamase inhibitors has received great interest as a method of combatting antibiotic-resistant *P. aeruginosa*, especially considering the declining productivity of the antibiotic discovery pipeline for treating Gram-negative infections [78,79]. Although these agents do not possess bactericidal effects alone against *P. aeruginosa*, when combined with a β-lactam, the inhibition of β-lactamases spares the co-prescribed β-lactam antibiotic from hydrolysis [118,119]. By the end of the 20th century, sulbactam and tazobactam (Figure 3) also reached clinical use, although these agents were primarily active against class A and D serine β-lactamases [120,121,122,123,124]. After nearly two decades of research, a novel class of β-lactamase inhibitors based on a bridged diazabicyclooctane (DBO) scaffold emerged, which includes inhibitors such as avibactam and relebactam (Figure 3) [125]. Other research efforts have similarly moved beyond β-lactam-based β-lactamase inhibitors, yielding boronates such as vaborbactam (Figure 3), which has been successfully paired with the carbapenem meropenem [126,127].

Avibactam, relebactam, tazobactam, and vaborbactam share similarities with regard to their inhibitory activities against the β-lactamases found in clinical *P. aeruginosa* isolates. Out of these four inhibitors, avibactam exhibits the broadest spectrum of activity, possessing the ability to target class A (e.g., TEM, SHV, CTX-M), class C (e.g., chromosomal AmpC), and some class D (e.g., OXA) β-lactamases [124]. Relebactam and tazobactam have been shown to target class A and C β-lactamases, although the latter is less effective than newer compounds, while vaborbactam has been shown to be particularly effective against class A KPC-type β-lactamases [124,128,129,130].

Based on prior epidemiological and in vitro evidence, 61.8–70.2% of β-lactam-resistant *P. aeruginosa* isolates were susceptible to ceftazidime/avibactam, with meropenem/vaborbactam exhibiting a narrower range of activity targeting 59.0% of isolates [131]. As imipenem/relebactam is a relatively new combination, data specific to β-lactam-resistant *P. aeruginosa* remain scarce. However, one study illustrated that the addition of relebactam failed to lower the minimum inhibitory concentrations (MICs) of β-lactam-resistant *P. aeruginosa* isolates below clinical breakpoints, with only 10.3% of tested isolates being classified as susceptible according to European Committee on Antimicrobial Susceptibility Testing criteria and 26.8% by Federal Drug Administration/Clinical and Laboratory Standards Institute criteria [132]. The development of new DBO-based inhibitors continues to be a major focus, with nacubactam (RG6080; Figure 3) and zidebactam (WCK-5107; Figure 3) [133,134] combinations having been shown to lower the MICs of β-lactams between 0.5 and 16 fold against *P. aeruginosa* isolates [135,136]. New boronates have also been explored, such as QPX7728 (Figure 3), an “ultrabroad” spectrum β-lactamase inhibitor that, when paired with meropenem, cefepime, or ceftolozane, demonstrated potent activity against >90% of all tested clinical *P. aeruginosa* isolates [137].

Despite the clinical success of β-lactamase inhibitors, it is no surprise to observe resistance emerging in MDR *P. aeruginosa* strains. Specifically, resistance against β-lactamase inhibitors in *P. aeruginosa* has occurred via mutations in the *ampC* gene or overexpression of efflux pumps. Against avibactam, amino acid substitutions such as E247K, G183D, T96I, and ΔG229-E247 in the AmpC β-lactamase significantly impair inhibitor binding while maintaining or enhancing catalytic activity [138,139]. Overexpression of the MexAB-OprM efflux pump has also been shown to facilitate expulsion of avibactam [139]. Tazobactam is similarly impacted by overexpression of the efflux pump MexXY-OprM, and a D245N mutation has been shown to drastically decrease the binding affinity of tazobactam to the *P. aeruginosa* β-lactamase PDC-315 [139,140]. Vaborbactam resistance has rarely been attributed to target modification-mediated resistance and often results from the production of an MBL, while resistance against relebactam has been documented to occur through the production of class A β-lactamases including GES-2, GES-19, and GES-20 [130,141]. Porin loss, including through the disruption of the *oprD* gene, has been observed to interfere with the entry of vaborbactam and relebactam as well [130,141].

### 3.2. Bacteriophages

First pioneered in the 1920s for the treatment of staphylococcal cutaneous furuncles and carbuncles [142], the initial development of phage therapy was largely carried out in the former Soviet Union during the 20th century with minimal penetrance into Western-dominated regions of medicine and research [143,144]. With the continued emergence of MDR bacteria, bacteriophages have been re-emphasized for use as potential synergistic agents in the treatment of MDR infections [145,146,147,148]. Although this therapy primarily uses lytic phages to kill the strains of bacteria that are causing an infection, an abundance of recent research has demonstrated that bacteriophages can also act synergistically and revive the bactericidal effects of antibiotics, including β-lactams.

**Figure 4:**
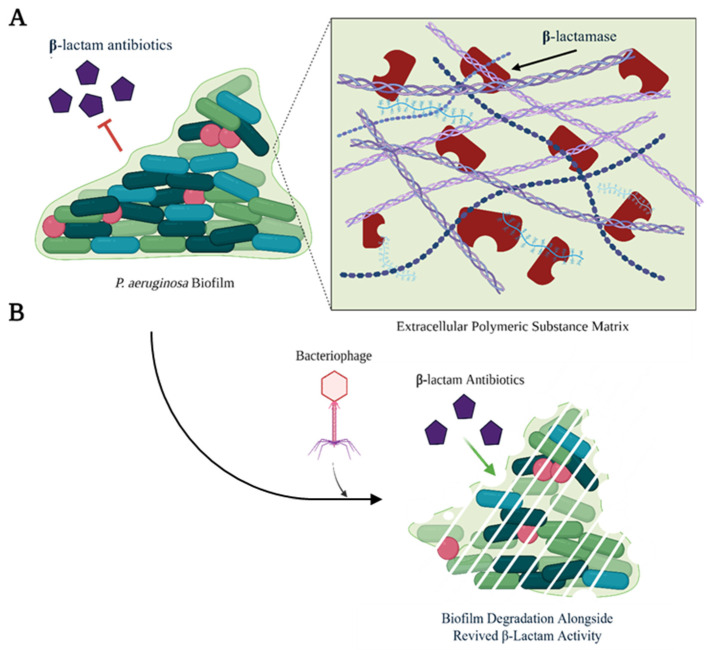
Synergistic use of bacteriophages with β-lactams against *P. aeruginosa.* (**A**) Biofilms produced by *P. aeruginosa* act as a physical barrier to β-lactams and serve as reservoirs for the accumulation of β-lactamases. These enzymes can inactivate β-lactam antibiotics before they enter the bacterial cells embedded in the biofilm. (**B**) Degradation of *P. aeruginosa* biofilms by bacteriophages can revive the efficacy of β-lactams by dispersing reservoirs of extracellular β-lactamases. Created in BioRender.

One proposed mechanism involves the destruction of biofilms, which are complex aggregates of bacteria encased in extracellular polymeric substances (EPS) that include polysaccharides (alginate, etc.), extracellular DNA, proteins, and lipids [149]. They contribute to β-lactam resistance by not only acting as a physical barrier to β-lactam entry, but also by forming an extracellular reservoir of β-lactamases (Figure 4A), as was observed by Dibdin et al. [150]. Specifically, increases in the concentration of extracellular β-lactamases depleted the concentration of active antibiotic, with alterations to the initial β-lactam concentration making no difference in the amount of active antibiotic remaining near the bacterial cells [150]. Wang et al. observed similar results where the pharmacodynamics of ceftazidime-induced lysis changed from a time-dependent model to a concentration model against biofilm-producing *P. aeruginosa*, when compared to a planktonic strain [151]. This shift towards a concentration model illustrates that the biofilm matrix can sequester and concentrate β-lactamase enzymes, thereby necessitating higher β-lactam concentrations to achieve bactericidal effects.

Bacteriophages can destroy biofilms by encoding or inducing the expression of enzymes that degrade the EPS that make up the matrix of a biofilm, or by inhibiting the synthesis of EPS (Figure 4B) [152]. For example, following treatment of *P. aeruginosa* PAO1 with non-bactericidal phages KT28 and KTN6, the rate of dye diffusion through a biofilm formed by this bacterium significantly increased, supporting the presence of a phage-encoded polysaccharide hydrolase able to digest glycosidic bonds found in biofilm-associated carbohydrate polymers [153]. An enzyme with similar activity was expressed by *P. aeruginosa* NCIMB 10548 following bacteriophage F116 infection, degrading over 75% of biofilm material after two hours of exposure [154].

Another study showcased that three phages (ATCC 12175-B1, ATCC 14203-B1, ATCC 14205-B1) produced an 85–95% reduction in *P. aeruginosa* PAO1 biofilm volume [155]. However, when surviving colonies were isolated and replated, an alginate-producing, slow-growing, mucoid strain was obtained. Sequencing revealed a mutation in the negative regulator of alginate production *mucA*, thereby conferring an alginate-hyperproducing phenotype notably characterized by an additional outer alginate capsular polysaccharide layer [156]. Further experimentation with the wild-type non-alginate producing PAO1 strain and the mucoid *mucA* strain yielded data consistent with this observation, whereby the presence of alginate completely abolished phage-dependent biofilm destruction, as the biovolume of wild-type PAO1 decreased by 96% while the biovolume of the mucoid strain remained unaffected. As with any form of antimicrobial intervention, this illustrates that *P. aeruginosa* can adapt and become less susceptible to biofilm-degrading phages.

Interestingly, alginate-degrading *P. aeruginosa*-specific bacteriophages have also been identified. In a study by Glonti et al., bacteriophage PT-6 rapidly reduced the viscosity of four different *P. aeruginosa* alginate polymers by 62–66% within 15 min [157]. This finding was supported by the detection of α,β-unsaturated uronic acid monomers that are produced by alginic acid polysaccharide depolymerases [158,159,160,161,162]. Given that mature *P. aeruginosa* biofilm development is dependent on quorum sensing, an engineered lactonase-encoding T7 bacteriophage was found to abolish *P. aeruginosa* biofilm formation by specifically degrading acyl homoserine lactone autoinducers [163]. Other studies using *P. aeruginosa*-specific bacteriophages have also reported similar reductions in biofilm formation, but their underlying mechanism of degradation remains unknown [162,164,165,166,167,168,169,170,171,172,173,174,175,176,177,178].

Despite the potential utility of biofilm-degrading bacteriophages against *P. aeruginosa*¸ a major limitation arises from the high level of specificity that occurs between phages and the targets that they recognize on the surfaces of host cells. Heterogeneity between clinical isolates of *P. aeruginosa*, particularly regarding outer membrane proteins and other surface molecules, can greatly impact phage infectivity and ability to produce biofilm-degrading enzymes. This necessitates the development of broader phage libraries, as well as robust and efficient methods for the preparation of personalized phage cocktails tailored to the particular strain(s) responsible for an infection.

Overall, the use of bacteriophages against *P. aeruginosa* can aid β-lactam therapy through the disruption of biofilms and the β-lactamase reservoirs they may contain that would otherwise prevent intact β-lactams from reaching their bacterial targets. This biofilm disruption could also enhance the ability of β-lactamase inhibitors to access bacterial cells, improving their potency in the context of an infection. Thus, integrating the use of bacteriophages with β-lactam/β-lactamase inhibitor combinations may provide a more comprehensive strategy to overcome β-lactamase-mediated β-lactam resistance in *P. aeruginosa*.

### 3.3. Antimicrobial Peptides

Antimicrobial peptides (AMPs), generally comprising 12–50 amino acid residues, exhibit a broad range of antibacterial activities, and are often produced by insects, birds, fish, and other animals [179]. In humans, AMPs are produced by immune cells during a front-line, innate immune response against invading pathogens. These AMPs are specific to bacteria, and their amphiphilic nature allows them to bind to negatively charged groups on the surfaces of bacterial cells, facilitating their entry into the periplasmic space and the cytoplasm [180,181]. Alongside their selective toxicity, thermal stability, and high water solubility, the ability of AMPs to disrupt the outer membranes of Gram-negative bacteria illustrates their significant potential to be used as adjuvant agents alongside β-lactam antibiotics [182,183].

**Figure 5:**
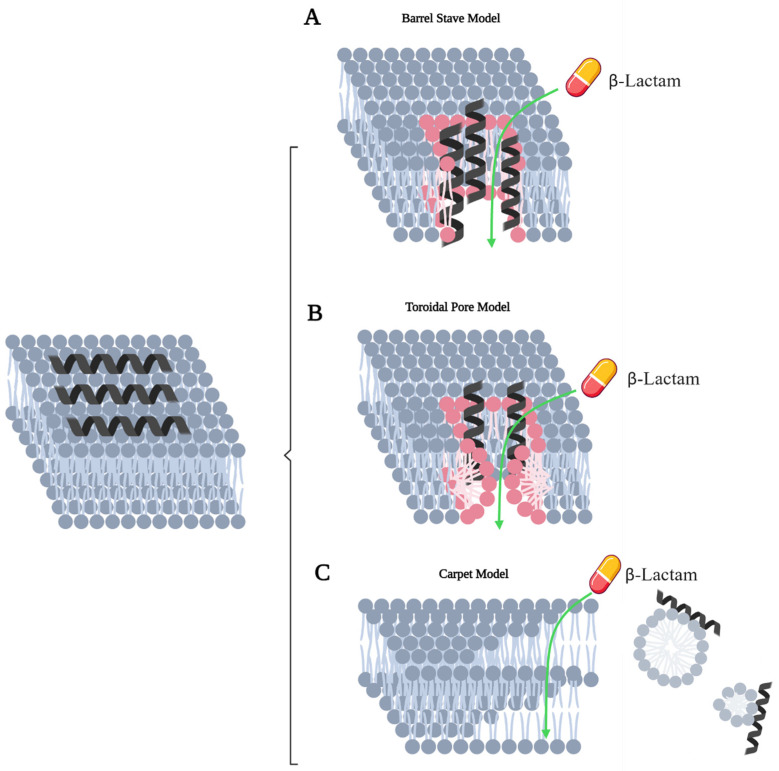
Mechanisms of AMP-mediated membrane disruption that allow for enhanced β-lactam entry. (**A**) In the barrel-stave model, the insertion of multiple AMPs into a membrane forms a protein-based transmembrane channel. (**B**) Although the toroidal pore model also involves the insertion of AMPs into the membrane, here, the peptides induce bending of lipid molecules such that a pore lined with lipid head groups is created. (**C**) The carpet model differs from the other two models described, as the AMPs primarily interact with the lipid head groups rather than inserting directly into the membrane; this interaction increases the permeability of the membrane and can cause the membrane to disintegrate and form micelles. Created in BioRender.

Although AMPs exhibit different bactericidal mechanisms, they can be broadly classified as agents that either directly target the bacterial membrane or that target other cellular components. AMPs that target the bacterial membrane remain selectively toxic, as the membranes of mammalian cells are primarily composed of net-neutral phospholipids (phosphatidylcholine, phosphatidylethanolamine, sphingomyelins) [184]. These membrane-targeting peptides are believed to act through three distinct mechanisms that have the potential to facilitate β-lactam entry across the outer membrane (Figure 5), as has been extensively reviewed elsewhere [185,186,187,188,189,190,191,192,193,194,195].

Specific to the use of AMPs as an adjuvant therapy against *P. aeruginosa*, researchers have observed synergistic activity between bacterial, synthetic, and animal-derived AMPs with β-lactam antibiotics. Although there is strong evidence for this synergism, the specific mechanism(s) by which these AMPs exert their synergistic effect is often not fully understood. Nonetheless, the studies described below demonstrate that structurally diverse AMPs from different origins are able to dramatically decrease the amount of β-lactam antibiotic needed to control the growth and biofilm formation of *P. aeruginosa*.

Jahanigirl et al. examined the effects of nisin and peptide P10 in combination with ceftazidime against colistin-resistant clinical isolates of *P. aeruginosa* [196]. Nisin is a bacteriocin produced by *Lactococcus lactis* that inhibits cell wall synthesis by binding and sequestering lipid II, a cell wall precursor necessary for peptidoglycan synthesis [194,197,198,199,200,201], while P10 is a synthetic derivative of mammalian LL-37 that induces bacterial cell wall lysis potentially through the barrel-stave, toroidal pore, or carpet models represented in Figure 5 [202,203,204]. When combined with ceftazidime, the MIC of P10 decreased between 2–8-fold across six *P. aeruginosa* isolates, with four illustrating synergistic effects (total fractional inhibitory concentrations (ΣFIC) < 0.5) [196]. A similar AMP, P5, was tested against the carbapenem-resistant *P. aeruginosa* M13513 strain [205] following confirmation of antimicrobial activity in previous studies [206,207]. When combined with meropenem at a concentration of half of the MIC, a 2-log decrease relative to individual treatments (i.e., P5 or meropenem alone) in a time-kill kinetics assay illustrated significant synergism [205]. Moreover, the ability of P5 to permeabilize the outer membrane of *P. aeruginosa* was demonstrated by the increased uptake of the fluorescent dye 1-N-phenylnapthylamine [205]. Although P5 did also exhibit biofilm-degrading properties, the potency of this activity was relatively low, as a reduction greater than 20% only occurred at P5 concentrations at least two-fold above the MIC [205].

Testing imipenem-resistant *P. aeruginosa* strains with decreased permeability arising from mutations to *oprD*, Rudilla et al. observed that treatment with 4 μg/mL of synthetic peptide AMP38 and 4 μg/mL of imipenem abolished bacterial growth compared to individual treatment groups [208]. The authors designed this peptide based on the structure of polymyxin, suggesting that AMP38 binds to lipopolysaccharides in the outer membrane of *P. aeruginosa*, displacing cations and inducing autolysis through membrane disruption [209]. Data from time-kill kinetics further supported the presence of synergistic activity, with all strains exhibiting an ΣFIC of less than 0.5 [208]. Interestingly, culturing *P. aeruginosa* PA11636 with AMP38 and imipenem decreased the minimal biofilm concentration from >500 μg/mL (imipenem alone) to 62.5 μg/mL, suggesting that AMP38 could contribute to β-lactam rescue in part through the destruction of biofilm-related β-lactamase reservoirs described above [208].

Contrasting the synthetic adjuvants described above, Shang et al. observed a novel, synergistic mechanism between tryptophan (Trp)-containing AMPs (L1W, L12W) and β-lactam antibiotics against the MDR *P. aeruginosa* MRPA0108 strain. When concentrations of L1W and L12W four-fold below their MICs were administered with ceftazidime and piperacillin, the authors observed an 8–32-fold and 2–12-fold reduction in the ceftazidime and piperacillin MICs, respectively [210]. Based on further transcriptomic analyses, the underlying synergistic mechanism was attributed to the L1W- and L12W-mediated downregulation of genes associated with β-lactam resistance, including the β-lactamase-encoding *ampC* gene and efflux pump genes *oprM*, *mexX*, and *mexA* [210]. L1W and L12W were also found to decrease biofilm formation by 33–53% by downregulating key genes involved in biofilm production (*pelA*, *algD*, and *pslA*) [210], illustrating the multifactorial nature of these Trp-containing AMPs in the context of the treatment of β-lactam-resistant *P. aeruginosa* infections.

Similar to the synthetic P5, P10, and AMP38 peptides, the ocellatin AMP family isolated from sebaceous secretions of the South American frog *Leptodactylus labyrinthicus* kills bacteria through a membrane-targeting mechanism [211]. When ocellatin PT3 was combined with ceftazidime against MDR *P. aeruginosa* isolates Pa1-SA2 and Pa4-SA2, Bessa et al. observed a 4–8-fold decrease in MIC with synergistic activity (ΣFIC < 0.5) [212]. Ocellatin PT3 was also able to inhibit biofilm formation in clinical isolate Pa4-SA2, rescuing β-lactam antibiotics by preventing the accumulation of β-lactamases in biofilms [212]. A study by Pandidan et al. further showed highly synergistic effects of melittin, an AMP in bee venom that forms toroidal pores [213], when combined with doripenem and ceftazidime against five *P. aeruginosa* isolates retrieved from burn patients [214]. The MIC of doripenem decreased between 32–128-fold when combined with melittin, demonstrating synergistic activity [214]. Synergism with ceftazidime was also reported across all five strains, but the MIC of ceftazidime decreased less relative to doripenem (4–64-fold reduction) [214].

Synthetic AMPs and those derived from natural sources show great promise as adjuvants that enhance the activity of β-lactam antibiotics against *P. aeruginosa* and other bacterial pathogens. However, *P. aeruginosa* can achieve high levels of β-lactam resistance by limiting the concentration of β-lactams in the periplasm through the simultaneous use of multiple resistance mechanisms (e.g., β-lactamase production, decreased permeability, increased efflux). Future studies could explore the extent to which AMPs impact the periplasmic concentration of β-lactams (and β-lactamase inhibitors) against *P. aeruginosa* strains that exhibit multiple resistance mechanisms. Potential synergy between AMPs and bacteriophages leading to increased biofilm disruption and outer membrane permeabilization would also be of interest. In addition, the mechanisms by which AMPs disrupt the outer membrane could be investigated further, supporting the rational development of more potent derivatives and providing insights into the propensity for bacterial resistance to develop.

## 4. Future Directions for Identification of β-Lactam Adjuvant Agents

Over the past few decades, the stagnation of antibiotic discovery has led to a steady decline in the number of approved antibiotics. As antibiotics cannot currently compete with the profitability of other blockbuster drugs (antihypertensive agents, etc.), many pharmaceutical companies, including Novartis, Sanofi, AstraZeneca, and GSK, have largely abandoned their “high risk” antibacterial programs [215,216]. Moreover, the recent bankruptcies of Aradigm, Achaogen, Tetraphase Pharmaceuticals, and Melinta Therapeutics, all of which had gained approval to sell novel antibiotics within the past decade, highlights how a shift towards identifying novel synergistic agents may help relieve the stress placed on the traditional antibiotic discovery pipeline [217]. Predicated on recent technological milestones, the emergence of high-throughput approaches to screen and identify lead synergistic compounds, particularly through artificial intelligence (AI) algorithms, may serve as a new foundation for antimicrobial discovery and treatment.

### 4.1. AI-Based Identification of Novel Synergistic Agents

Several papers have been written on the development of training algorithms and the current and future projections of AI-based identification of novel antimicrobial agents [218,219,220,221,222]. Above all, these manuscripts highlight a revolutionary advantage AI has over traditional, manual analyses of natural and synthetic molecular libraries—the capacity of AI to generate potential novel antibacterial agents beyond our current knowledgebase of existing compounds simply cannot be matched. A recent example involves the identification of the antibiotic abaucin for the treatment of MDR *Acinetobacter baumannii* through a mechanism that involves the disruption of lipoprotein trafficking [223]. However, this section will focus on summarizing published examples specific to the design and development of novel agents that may have synergistic activity with β-lactams.

AI has been applied to the identification of all three categories of adjuvants described in this review, as demonstrated by the following examples. In relation to identifying novel β-lactamase inhibitors, Parvaiz et al. utilized Site Identification by Ligand Competitive Saturation technology to generate pharmacophore models and functional group requirements for the β-lactamase CMY-10 active site [224]. Subsequent machine learning-based random forest methods were then used to screen and filter a repository of 700,000 compounds, whereby 74 were subjected to in vitro β-lactamase inhibition assays [224]. Of these, 11 were demonstrated to inhibit CMY-10, with one compound showing significant synergistic activity with the cephalosporin cefixime against MDR clinical isolates of *Enterobacter cloacae*, *E. alvei*, and *E. agglomerans* [224]. A search for related inhibitors yielded an additional 28 compounds, many of which also exhibited synergistic activity against MDR isolates [224].

Focusing on the identification of novel bacteriophages, poor-quality annotations of phage genomes from environmental sources hamper the efficient production of species-specific phages, including those that target *P. aeruginosa* [225,226]. To tackle this issue, Thung et al. developed STEP^3^, a computational tool that identifies, distinguishes, and categorizes genomic features in uncharacterized phages [227]. As structural motifs on the phage tail facilitate binding to the bacterial surface, STEP^3^ can be used to classify proteins with conserved features for researchers to easily pinpoint the target species of a given phage [227]. The integration of data into an ensemble framework also remains robust despite the high evolutionary rates of phage proteins, with prediction accuracy significantly surpassing traditional pairwise sequence matching methods of phage protein identification [228].

In the field of AMPs, Bhadra et al. assessed the accuracy of 19 random forest algorithms in predicting the antibacterial and synergistic activity of unknown peptides (166,791 sequences) based on the amino acid sequences of classified AMPs (3268 sequences). Following their analyses, the model AmPEP produced an accuracy score of 96% and an Area Under the Receiver Operating Characteristic Curve of 0.99, illustrating the high discriminatory power of the model in differentiating hits from false positives in the context of discovering antibacterial AMPs [229]. However, the study did not validate the results through in vitro growth/time-kill kinetics analyses. Nonetheless, with the rapid advancement of algorithms and learning models as illustrated by the three examples presented above, it will be no surprise to witness a significant integration of AI into the framework of antimicrobial drug discovery.

### 4.2. Challenges Moving Forward

Although the rise of AI-based identification algorithms may serve as a nearly limitless reservoir of new lead compounds for the development of novel synergistic agents and beyond, several roadblocks remain from development to commercialization. Aside from the immense financial burden of drug development, many lead compounds that show promise in in vitro studies are ineffective when tested in vivo. Challenges with reproducibility have also been extensively noted between institutions [230,231,232,233]. As with any synergistic pharmaceutical intervention, ensuring that the pharmacological profile of the combination has no unfavorable outcomes or off-target effects remains a major hurdle. For example, many β-lactam/β-lactamase inhibitor combinations identified in vitro require high concentrations for clinical efficacy, potentially leading to toxicity or other undesired outcomes [234]. Reports of antibacterial and synergistic agents targeting both bacterial and human pathways further illustrate the importance of preclinical screening [235]. Surveying for potential pharmacological contraindications also remains a priority. For example, reserpine, a known synergistic agent to norfloxacin, was shown to directly act as a calcium antagonist on mammalian smooth muscle cells [236]. Compounds in a combinatory formulation may also exhibit differing pharmacodynamics and kinetics, including dissimilar methods of absorption, metabolism, and excretion. In these cases, more work may be required to structurally modify a specific agent in favor of similar pharmacological profiles. For instance, the β-lactamase inhibitor sulbactam in its native form exhibited poor oral absorption before a pivaloyloxymethyl ester functional group was introduced [237].

Beyond these pharmacological challenges, AI-based methods for drug discovery have several inherent limitations. A primary concern lies in model generalizability, as the use of incomplete, inconsistent, or low-quality data to train an algorithm can significantly compromise its performance. For instance, a model may perform poorly when predicting the mechanism of a novel compound if limited or biased training datasets are used. Additionally, while AI models can rapidly identify candidate compounds, validation through in vitro or in vivo experiments is essential, as algorithms may not effectively account for the complex interactions that occur between a pharmaceutical, a patient, and a pathogen. As the integration of AI into biomedical research continues to develop at an incredible pace, its incorporation into existing regulatory frameworks can lag behind, complicating the clinical development pathway and regulatory approval. These limitations, combined with increasing computational resource needs and biases toward known chemical scaffolds, can stifle the discovery of truly novel agents. Addressing these limitations will be crucial to fully realize the potential that AI offers towards the discovery of β-lactam adjuvants and other synergistic antimicrobial agents.

Above all challenges, however, novel synergistic agents still exert a selective pressure against bacteria and are thus not immune to the development of resistance. Relevant to β-lactamase inhibitors, strains harboring mutations that modify the active sites of β-lactamases are naturally favored when inhibitors are used. As one example of many, Alonso-Garcia et al. documented the presence of mutations in the β-lactamase gene *bla*_PDC,_ which contributed to resistance against cephalosporin/β-lactamase inhibitor combinations and imipenem/relebactam for a *P. aeruginosa* strain that was responsible for nosocomial meningoventriculitis [139]. Similarly, bacteriophages may be evaded through mutations to genes that encode for bacterial surface proteins, and by the CRISPR/Cas-9 system, which prevents integration of viral DNA during the lysogenic cycle and disrupts viral genome replication and protein production during the lytic cycle [238]. AMPs are not immune either, with studies demonstrating how modifications to cell surface structures (e.g., increased expression of anionic capsular polysaccharides) in *P. aeruginosa* confer resistance [239,240,241].

## 5. Conclusions

In conclusion, the exploration of synergistic agents, particularly in tandem with β-lactam antibiotics, marks a critical stride in the ongoing battle against resistant *P. aeruginosa*. The clinical success of β-lactamase inhibitors is perhaps the greatest and most recent example of this feat, offering novel therapeutic options against β-lactam-resistant strains. While the integration of artificial intelligence offers unprecedented opportunities for identifying novel compounds, the constant threat of resistance, in tandem with financial hurdles and intricate pharmacological considerations, emphasizes the need for cautious optimism. Future work should therefore not only be focused on developing novel antibiotics and synergistic agents but must also continue to address and improve antibiotic stewardship in clinical and agricultural settings.

## Figures and Tables

**Table 1:** Overview of β-lactamase classification and activity.

Ambler Class	Representative Members Found in *P. aeruginosa*	Substrate Scope and Relevance
A (SBLs)	KPC-2, GES-2	Certain members of this class can degrade all β-lactam subclasses; includes ESBLs and carbapenemases [44]
B (MBLs)	NDM-1, IMP-1, VIM-2	Most noted for carbapenemase activity; some members degrade penicillins and cephalosporins; do not protect against monobactams [45]
C (SBLs)	AmpC, PDC-1	Generally effective against penicillins and cephalosporins; some have ESBL activity; limited ability to degrade carbapenems [46]
D (SBLs)	OXA-10, OXA-48	Degrade certain penicillins and cephalosporins; some members have ESBL or carbapenemase activity [47]

**Table 2:** Efflux pump-mediated β-lactam resistance in *P. aeruginosa*.

Efflux System	β-Lactam Substrates
MexAB-OprM	All tested β-lactams, * excluding imipenem [104,114]
MexXY-OprM	All tested β-lactams, * excluding carbenicillin, sulbenicillin, cefsulodin, ceftazidime, moxalactam, flomoxef, aztreonam, and imipenem [104]
MexCD-OprJ	All tested β-lactams, * excluding carbenicillin, sulbenicillin, cefsulodin, ceftazidime, moxalactam, aztreonam, and imipenem [104]
MexEF-OprN	Imipenem [115,116]

* Penicillin G, cloxacillin, nafcillin, amoxicillin, piperacillin, carbenicillin, sulbenicillin, cefamandole, cefuroxime, cefoperazone, cefotaxime, ceftizoxime, ceftriaxone, cefsulodin, ceftazidime, cefpirome, cefepime, cefozopran, cefoselis, cefoxitin, moxalactam, flomoxef, imipenem, and meropenem.

## Data Availability

No new data were created or analyzed in this study. Data sharing is not applicable to this article.

## Abbreviations

The following abbreviations are used in this manuscript:

MDR	Multidrug resistant
PBP	Penicillin-binding protein
SBL	Serine β-lactamase
MBL	Metallo-β-lactamase
ESBL	Extended-spectrum β-lactamase
RND	Resistance-nodulation-division
DBO	Diazabicyclooctane
MIC	Minimum inhibitory concentration
EPS	Extracellular polymeric substances
AMP	Antimicrobial peptide
FIC	Fractional inhibitory concentration
Trp	Tryptophan
